# Assessing the Effect of Litter Species on the Dynamic of Bacterial and Fungal Communities during Leaf Decomposition in Microcosm by Molecular Techniques

**DOI:** 10.1371/journal.pone.0084613

**Published:** 2013-12-19

**Authors:** Wenjing Xu, Lingling Shi, Onchim Chan, Jiao Li, Peter Casper, Xiaoming Zou

**Affiliations:** 1 Key Laboratory of Tropical Forest Ecology, Xishuangbanna Tropical Botanical Garden, The Chinese Academy of Sciences, Kunming, Yunnan, China; 2 University of Chinese Academy of Sciences, Beijing, China; 3 Institute of Agricultural Engineering Anhui Academy of Agricultural Sciences, Hefei, Anhui, China; 4 Department of Biology, Faculty of Life Sciences, Yunnan University, Kunming, Yunnan, China; 5 Department of Experimental Limnology, Leibniz-Institute of Freshwater Ecology and Inland Fisheries, Stechlin, Germany; 6 College of Forest Resources and Environment, Nanjing Forestry University, Nanjing, Jiangsu, China; 7 Institute for Tropical Ecosystem Studies, University of Puerto Rico, San Juan, Puerto Rico, United States of America; University of Kentucky College of Medicine, United States of America

## Abstract

Although bacteria and fungi are well-known to be decomposers of leaf litter, few studies have examined their compositions and diversities during the decomposition process in tropical stream water. Xishuangbanna is a tropical region preserving one of the highest floristic diversity areas in China. In this study, leaf litter of four dominant plant species in Xishuangbanna was incubated in stream water for 42 days during which samples were taken regularly. Following DNA extraction, PCR-DGGE (denaturing gradient gel electrophoresis) and clone-sequencing analyses were performed using bacterial and fungal specific primers. Leaf species have slightly influences on bacterial community rather than fungal community. The richness and diversity of bacteria was higher than that of fungi, which increased towards the end of the 42-day-incubation. The bacterial community was initially more specific upon the type of leaves and gradually became similar at the later stage of decomposition with alpha-proteobacteria as major component. Sequences affiliated to methanotrophs were obtained that indicates potentially occurrence of methane oxidation and methanogenesis. For the fungal community, sequences affiliated to *Aspergillus* were predominant at the beginning and then shifted to *Pleosporales*. Our results suggest that the microorganisms colonizing leaf bioﬁlm in tropical stream water were mostly generalists that could exploit the resources of leaves of various species equally well.

## Introduction

 Allochthonous leaf litter inputs are the main carbon and energy sources for headwater streams of forests [1,2]. Along with driving decomposition processes, fungi and bacteria are important intermediaries in energy flow in stream ecosystems, as they can break down large molecules, such as cellulose, chitin, and lignin, into smaller compounds that can be taken up by the biota of higher trophic levels [3,4]. Fungi are generally more efficient than bacteria in exploiting available resources through invasion and enzymatic hydrolysis of leaf material and lysed hyphae [5-7]. Consequently diversity of fungi and their decomposition capabilities during leaf litter decomposition have been investigated from the various studies [8]. Bacteria can benefit from the compounds released by this process and take advantage of the increased surface area provided by the macerated plant tissue (through fungal action) and fungal hyphae for colonization [9]. It is suggest that the role of bacteria in leaf decomposition has been underestimated. Different groups of bacteria and fungi have various biochemical and physiological capabilities, and these differences may influence the leaf decomposition process that underscores the need to examine microbial community structure.

 The vast majority of leaf decomposition studies were conducted in temperate area and less was known about the importance of microbial processing in tropical systems [10,11]. Compared to temperature forest, tropical ecosystem harbored relatively high species richness of riparian forests and result in a greater complexity of leaf litter composition [12]. The chemical composition of plant litter is commonly considered to indicate its quality as a resource for decomposer organisms. Many studies have shown that microbial communities are differentially abundant and structure according to types of leaf species or substrata in tropical forests [13-15]. The relative importance of bacteria and fungi in leaf decomposition and invertebrate feeding may be influenced by the chemical composition of leaves [16,17]. Nevertheless, no studies in tropical areas have focused on understanding the ecological significance of such ‘preferences’ by decompose.

Assessment of the relative importance of both fungi and bacteria in lotic food webs is ongoing but incomplete, because most of them were base on cultural dependence techniques. Fungal communities on decomposing leaf litter have traditionally been studied by counting conidia, which are limited to aquatic hyphomycetes and require the induction of sporulation [18]. Bacterial community analyses relied primarily on culturing- based techniques although many taxa have not been successfully cultured. Recent advances in molecular taxonomy offer a new approach for analyzing community structure for both fungi and bacteria [19]. DNA profiles are generally quicker to generate than morphological profiles, do not require sporulation or culturing, and can detect all life stages of microbes even when populations are small [20,21]. Comparisons of morphological and molecular approaches indicate that diversity is underestimated by morphology. Molecular tools have revealed large spatial and temporal variation among microbial communities and more subtle variation among substrate types within a habitat. 

Microcosm experiments have been found to be useful for testing factors that influence decomposition under controlled conditions [22,23]. In this study, we used a microcosm experiment to test the role of litter species on microbial decomposition incubation. Four dominant plant species, that have various C/N ratios, were selected to elucidate the effect of leaves on the communities. For this reason, DGGE (denaturing gradient gel electrophoresis) and clone-sequencing methods were used to analyze the PCR amplicons of the small subunit of rRNA gene of bacteria and fungi. We predicted that community structure and diversity would also differ among the four litter species based on the differences of chemical composition described above. Specially, we aimed to explore (1) the effect of leaf species on the changes of microbial communities (2) the differences between fungal and bacterial diversity patterns and community composition.

## Materials and Methods

### Sample collection and leaf litter incubation

Leaf litter of representative dominant plant species were collected from the ground of a rain forest in the watershed of Mane, Menglun, Xishuangbanna, SW China in April 2007 at the end of dry season of peak leaf litter fall. The leaves were air-dried and subjected to total carbon, nitrogen and phosphorous measurements. Leaf litter of three plant species *Baccaurea ramiflora Lour*. ( *Baccaurea ramiflora*), *Pleioblastus amarus* (Keng) keng (*Pleioblastus amaru*) and *Pometia tomentosa* (Bl.) Teysm. et Binn. (*Pometia tomentosa* ) that have various C: N ratios were selected for this study. In addition, leaf litter of *Hevea brasiliensis* (Willd. ex A. Juss.) Muell. Arg. (*Hevea brasiliensis* ) from a neighboring watershed of rubber tree plantation was sampled. Water from a headwater stream in the rain forest was collected for incubation experiment and the water properties were measured. The samples collecting in field have got permission from Xishuangbanna Station for Tropical Rain Forest Ecosystem Studies (XSTRE).

Total carbon, nitrogen and phosphorous of the leaf litter were examined by chromic acid wet oxidation, Auto Kjeldahl Unit model K370 (Buchi, Flawil, Switzerland) and inductively coupled plasma-atomic emission spectrometry (ICP-AES, model IRIS Advantage-ER, TJA, Franklin MA, USA), respectively. Conductivity, pH, dissolved oxygen and temperature of the stream water were measured by conductivity meter (Hach SensIon5, Colorado, USA), pH meter (PHS-3C, Shanghai Precision & Scientific Instrument, Shanghai, China) and oxygen meter (YSI 550A, Yellow Springs Instrument Co., Ohio, USA), respectively. Total organic carbon and total inorganic carbon concentrations of the water were examined by a TOC analyzer (Elementar Systeme liquiTOCII, Hanau, Germany). Nitrate, ammonium, total phosphorous and soluble reactive phosphorous were analyzed spectrophotometrically following Water Quality, Standard Methods of P. R. China (1987). All measurements were performed in replicates.

Incubation was conducted in series of sterilized 1L flasks in July 2007. Approximately 0.5 g of the leaf litter was placed in each flask containing 0.8 L of stream water. The incubation was control under room temperature. The flasks were aerated with continuous bubbling from the bottom of the flasks to ensure aerobic conditions. The air for bubbling has gone through sterile cotton before entering bottles to limit contamination. Leaf litter was harvested on days 1, 3, 7, 14, 28 and 42. The incubation was replicated two times. Sampled leaf litter was frozen at -20°C prior to microbial community structure analyses. All samples were examined by DGGE analysis. For clone sequencing, only leaf litter samples of days 1 and 42 were analyzed and equal weights of sub-sample replicates from incubations were pooled for analysis.

### DNA extraction and PCR amplification

Total DNA was extracted following Nikolcheva & Barlocher (2004) [24]. In brief, 0.5 g leaf litter was grounded briefly in liquid nitrogen, and then MoBio Soil DNA Extraction Kit was used following the manufacturer’s instructions. The extracted DNA was amplified by the primer sets 357fGC (5’end incorporated with a 40 bases GC-rich sequence) and 907r targeting the 16S rRNA gene for analysis of bacterial community by DGGE. For clone sequencing, the same primer set without GC clamp was used. For fungal community, NS1-GCfung [20] and NS1-fung (without GC clamp) was used to amplify the 18S rRNA gene region for DGGE analysis and clone sequencing, respectively.

### DGGE analysis

DGGE analysis was performed as described by Muyzer (1998) using a BioRad DCode system [21]. For each sample, 400 ng of the PCR amplicons were loaded. 6% acrylamide gel with denaturing gradient of 40%-60% was used for analysis of bacterial community and 8% gel with 30%-40% was used for fungal community. The DGGE was conducted at 80V, 60°C for 12 hours. The profiles obtained were analyzed by QuantityOne (BioRad) and standardized according to Dunbar et al. (2009) [25]. The standardized relative intensities of the DGGE bands were subjected to cluster analysis based on Euclidean distances of unweighted pair-group method arithmetic mean, UPGMA (Multi Variate Statistic Package, MVSP version 3.1, Kovach Computing Services). Richness was reflected as the number bands detected. Shannon diversity index was calculated regarding each band as a single ribotype and the standardized band relative intensity as relative abundance. Paired sample T test was used to evaluate whether significant difference exists between samples.

### Cloning and sequencing analyses

The PCR amplicons were purified by agarose gel extraction (BioAsia, Shanghai, China), and then were cloned using pMD19-T vector (TaKaRa D102A, Takara Bio, Otsu, Japan) following the manufacturer’s instructions. Clones were screened by blunt-white plate and PCR using vector primers M13 (Sangon, Shanghai, China). Cloned sequences were determined using an ABI 3730 sequencer (Applied Biosystems, Foster City, USA) at BioAsia Biotechnology Ltd., Shanghai, China. The sequences obtained were aligned and their phylogenetic relationship was determined as described in Chan et al. [26]. Phylogenetic trees based on aligned sequences of 800 and 250 bases, respectively, for bacteria and fungi were constructed using 100-fold bootstrap analysis by neighbor-joining and parsimony algorithms using the Phylogenetic Inference Package (PHYLIP) version 3.6. The clone sequences generated in this study were deposited in the GenBank database under the accession numbers. The constructed neighbour-joining trees were subjected to UniFrac (http://bmf2.colorado.edu/unifrac/index.psp) to test if significant differences exist between individual environmental samples using UniFrac distance metrics and the P test algorithms.

## Results

### Characteristics of leaf litter and stream water

Leaf litter of the plant species *Pleioblastus amarus* has the lowest C/N ratio (26.7) and *Pometia tomentosa* has the highest C/N (54.7) among the four studied representative dominant species in Xishuangbanna (Table 1). Leaves from rubber trees *Hevea brasiliensis* have one of the higher C/N ratios (46.1). The properties of the sampled stream water were as follows: temperature 20°C, pH 7.8, dissolved oxygen 7.4 mg l^-1^ (80% saturation, elev. 600 m), conductivity 406 μs cm^-1^, total organic carbon 7.6 mg l^-1^ TOC-C, total inorganic carbon 23.5 mg l^-1^ TIC-C, nitrate 0.18 mg l^-1^ NO_3_^-^-N, ammonium 0.06 mg l^-1^ NH_4_^+^-N, total phosphorous 0.037 µg l^-1^ TP-P and soluble reactive phosphate 0.035 µg l^-1^ PO_4_^-^-P.

**Table 1:** Total carbon, nitrogen and phosphorous contents of leaf litter of four representatives dominant plant species in Xishuangbanna.

	Total carbon (mg/g-dry-wt)	Total nitrogen (mg/g-dry-wt)	Total phosphorous (mg/g-dry-wt)	C/N
*Baccaura ramiflora L*	541.0±27.6	13.7±0.8	0.81±0.08	39.4
*Hevea brasiliensis*	515.0±14.8	11.2±0.2	0.38±0.03	46.1
*Pleioblastus amarus*	427.2±23.9	16.0±0.1	0.81±0.05	26.7
*Pometia tomentosa*	469.7±4.2	8.6±0.2	0.46±0.03	54.7

Average values and standard deviations of three replicates are indicated. C/N ratios are calculated from the average values.

### DGGE profiles of bacterial communities

**Figure 1:**
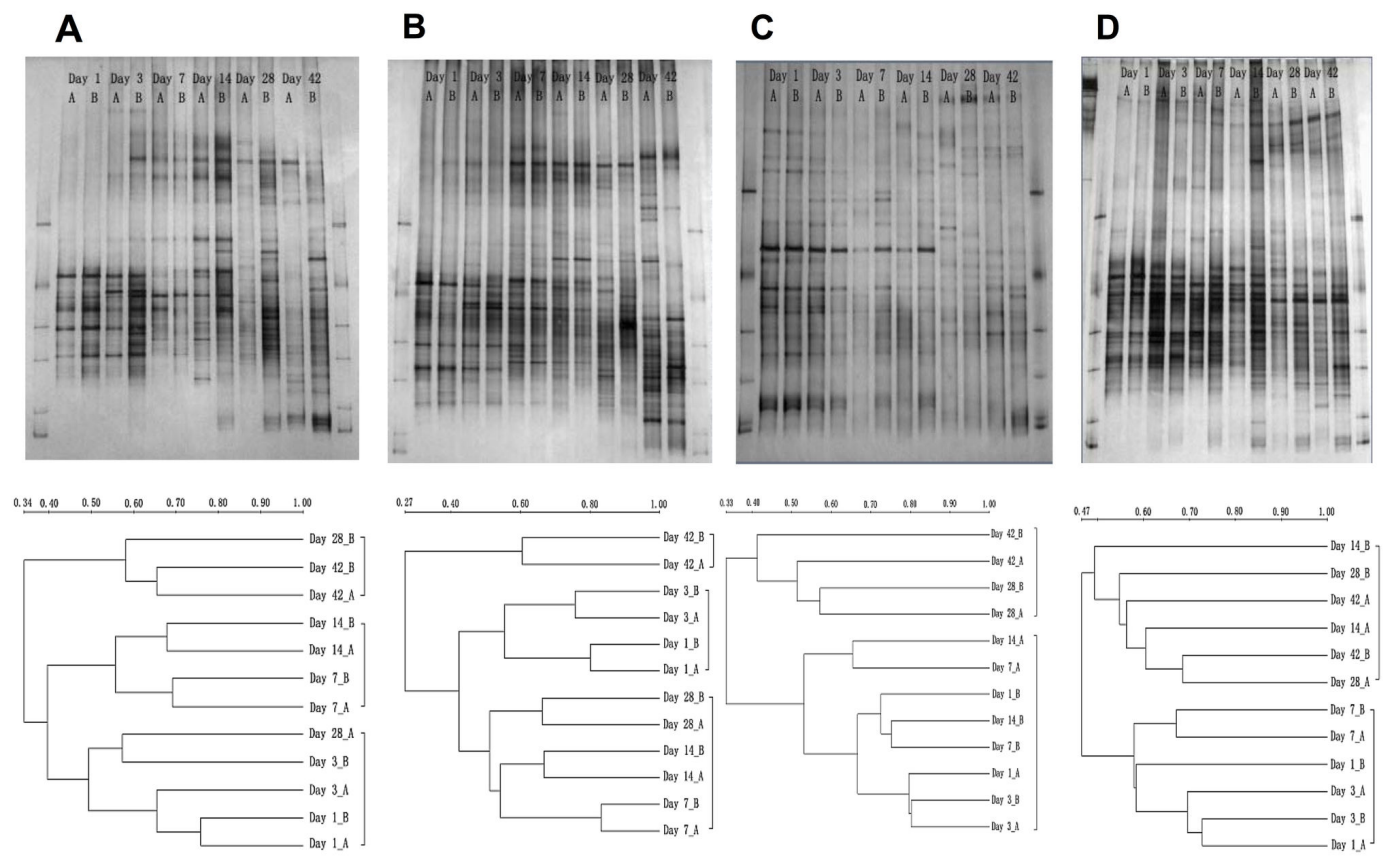
DGGE profiles of bacterial 16S rRNA gene amplicons derived from the leaf litter of the plant species A: *Baccaure ramiflora*, B: *Hevea brasiliensis*, C: *Pleioblastus amarus* and D: *Pometi tomentos*. The numbers of days of incubations are indicated. Each sampling day has two replicates of separate incubations denote by A and B. The left- and right-most lanes are markers.

**Figure 2:**
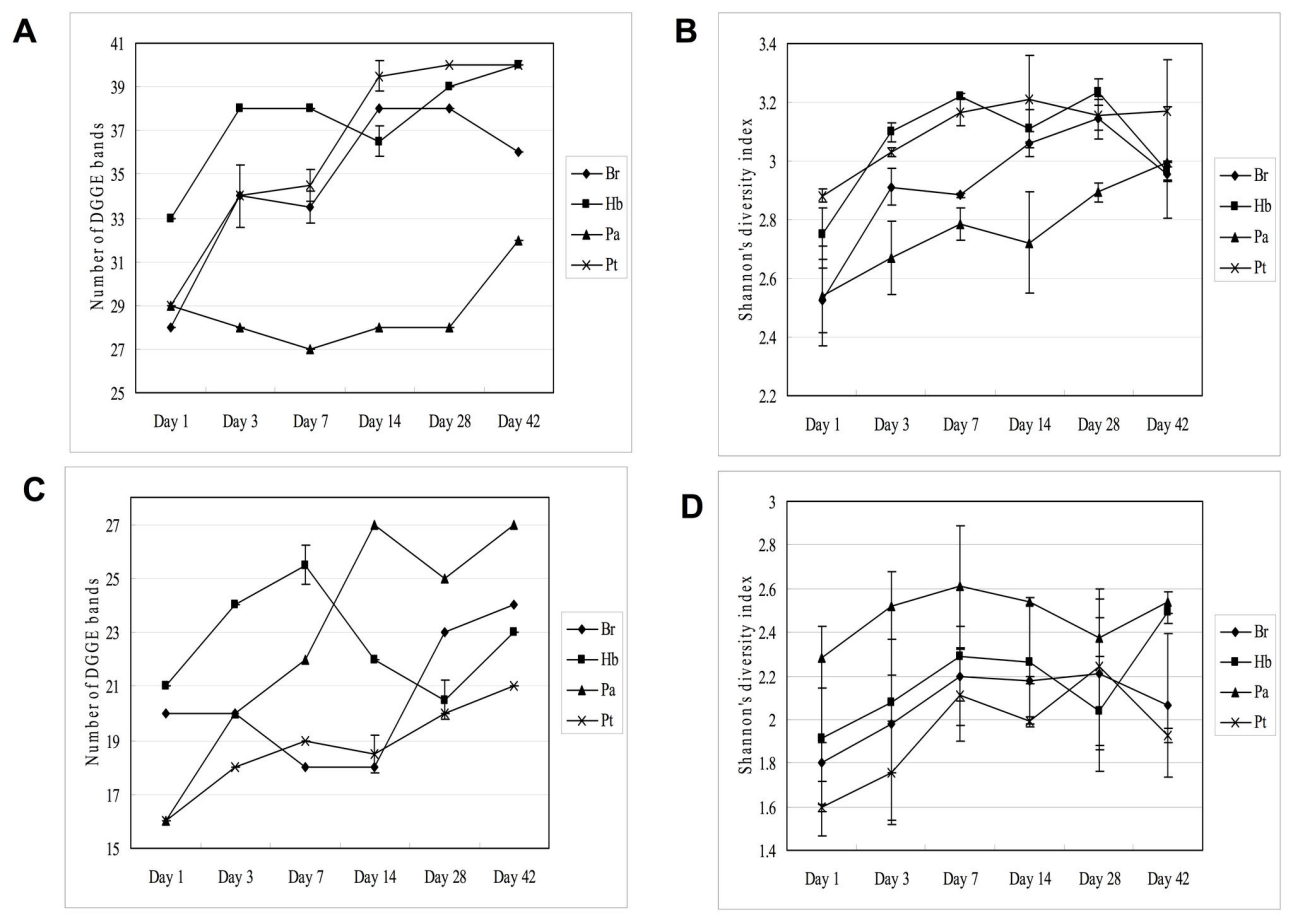
Richness and diversity of microbial communities of the leaf litter of 4 plant species during 42 days incubation A: number of bands obtained from DGGE analysis of bacterial 16S rRNA gene PCR amplicons, B: Shannon’s index calculated from bacterial DGGE profiles, C: number of bands obtained from DGGE analysis of fungal 18S rRNA gene PCR amplicons, and D: Shannon’s index calculated from fungal DGGE profiles. The plant species *Baccaure ramiflora, Hevea brasiliensis, Pleioblastus amarus* and *Pometi tomentos* were denoted by Br, Hb, Pa and Pt, respectively.

Twenty-seven to 40 bands were detected from each sample by DGGE analysis of the PCR amplified bacterial 16S rRNA gene fragments (Figure 1 and 2A). Three stages, 1-3 days, 7-28 days and 42 days of incubation, could be classified for the leaves of *Baccaura ramiflora* L. and *Hevea brasiliensis* (Figure 1A and B), and two stages for *Pleioblastus amarus* and *Pometia tomentosa* (Figure 1C and D). The richness of the bacterial communities that reflected by the number of bands increased remarkably over time during the incubation of leaves of all four plant species (Figure 2A), with DGGE bands distributed over a wider denaturing gradient at the later stage of incubation. In general, the diversity reflected from Shannon’s index also increased (Figure 2B).

### Clone sequences of bacterial communities

Nearly 40 sequences of bacterial 16S rRNA gene clones were obtained from each of the leaf litter species at the first and the last day of incubation (Table 2, phylogenetic trees not shown). All samples comprised of >50% of Proteobacteria. In the first day, the bacterial composition was dominated by beta- and/or gamma-Proteobacteria that together accounted for 66% (*Pometia tomentosa*) to 100%( *Hevea brasiliensis*) of all sequences obtained. At day 42, alpha-Proteobacteria became predominant, which contributed to >49% of each clone library, except the leaf litter from *Pleioblastus amarus*. Various bacterial classes or phyla including beta-Proteobacteria, gamma-Proteobacteria, Actinobacteria and Bacteriodetes were distributed more evenly in the bacterial community of *Pleioblastus amarus*, but alpha-Proteobacteria (19%) were still the majority. Shannon’s diversity index of bacteria in the phylum level of all four leaf species increased at the end of incubation.

**Table 2:** Relative abundance and diversity of bacterial 16S rRNA gene fragment sequences in various taxa.

	Baccaurea ramiflora		Hevea brasiliensis		Pleioblastus amarus		Pometia tomentosa	
	Day 1	Day 42	Day 1	Day 42	Day 1	Day 42	Day 1	Day 42
Proteobacteria	97	62	100	69	82	59	89	79
Alpha Proteobacteria	5	51	n.d.	49	10	19	24	67
Novosphingobium	3	3	n.d.	18	5	5	8	33
Beta Proteobacteria	59	3	16	5	51	16	5	5
Massilia	n.d.	n.d.	n.d.	n.d.	41	n.d.	n.d.	n.d.
Duganella	10	n.d.	n.d.	n.d.	n.d.	n.d.	n.d.	n.d.
Aquabacterium	23	n.d.	n.d.	n.d.	n.d.	n.d.	n.d.	n.d.
Acidovoras	10	n.d.	3	n.d.	n.d.	n.d.	n.d.	n.d.
Gamma Proteobacteria	33	n.d.	84	10	21	19	61	5
Acinetobacter	23	n.d.	22	10	n.d.	n.d.	40	n.d.
Enterobacter	10	n.d.	12	n.d.	15	n.d.	5	n.d.
Aeromonas	n.d.	n.d.	19	n.d.	n.d.	n.d.	8	n.d.
Pseudomonas	n.d.	n.d.	31	n.d.	n.d.	3	5	n.d.
Delta Proteobacteria	n.d.	5	n.d.	5	n.d.	5	n.d.	3
unclassified Proteobacteria	n.d.	3	n.d.	n.d.	n.d.	n.d.	n.d.	n.d.
Actinobacteria	n.d.	3	n.d.	15	8	16	n.d.	3
Chloroflexi	n.d.	19	n.d.	3	n.d.	5	n.d.	n.d.
Bacteriodetes	3	8	n.d.	n.d.	8	14	5	15
Planctomycetes	n.d.	3	n.d.	5	n.d.	n.d.	n.d.	n.d.
Acidobacteria	n.d.	n.d.	n.d.	5	n.d.	n.d.	n.d.	3
Verrucomicrobia	n.d.	n.d.	n.d.	n.d.	n.d.	3	n.d.	n.d.
Spirochaetes	n.d.	n.d.	n.d.	n.d.	n.d.	n.d.	3	n.d.
unclassified Bacteria	n.d.	5	n.d.	3	3	3	3	n.d.
Number of clones obtained	39	37	32	39	39	37	38	39
Shannon’s diversity index (based on phyla)	0.12	1.17	0	1.04	0.65	1.23	0.45	0.66
Shannon’s diversity index (based on 97% sequence similarity)	2.27	3.12	2.21	3.09	2.67	3.08	2.21	2.65

Bacterial taxa that were not detected in the clone library are denoted as n.d. Diversity is calculated based on bacterial phyla and 97% sequence similarity as operational taxonomy unit.

At the beginning of decomposition, leaf litter was inhabited by bacteria clustered specifically to certain phylogenetic taxa. Sequences affiliated to the genera Acinetobacter, Enterobacter and Aeromonas in gamma-Proteobacteria comprised to 40% of the total bacterial clones of the four leaf species of day 1. Leaf litter from *Pleioblastus amarus* had only 15% of gamma-Proteobacteria, but exclusively exhibited 41% of Massilia, beta-Proteobacteria. In addition, *Baccaura ramiflora* L and *Hevea brasiliensis* further presented distinct clusters of Aquabacterium and Pseudomonas, respectively. The sequences within these clusters had high similarity, all clusters had >95% sequence similarity and 85% sequences had >97% similarity.

The bacterial compositions were more diverse after incubation of leaves for 42 days. The only exception was *Pometia tomentosa*, which comprised a *Novosphingobium*-clade consisting of one-third of its bacterial sequences with >95% similarity. Other alpha-Proteobacteria that obtained from the leaf litter samples at day 42 were Rhizobiaceae, Bradyrhizobiaceae, Rhodobacteriales, Hyphomicrobiaceae, Caulobacteriales and Type II methanotrophs. The finding of sequences affiliated to the genera *Methylophilus* and *Methylibium*, beta-Proteobacteria, and to anaerobic bacteria *Levilinea* indicates that methane-oxidation and methanogenesis processes might occur in the leaf litter at a latter stage of leaf litter decomposition in the stream water. Other relatively major bacterial clusters were Actinomycetales, Crenotricaceae (Chitiophaga and Terrimonas) and Herpetosiphon. Regarding sequence similarity of >97% as a species or an operational taxonomy unit, Shannon’s diversity index also increased at the end of incubation. Both UniFrac distance metrics and P tests of the phylogenetic trees showed significant differences (P≤0.01) between incubation days 1 and 42 for leaf litter of all plant species. Marginal differences (P≤0.05) were found among the bacterial communities of the four leaf litter species at the beginning of incubation. Differences among various leaf species were not significant after incubation for 42 days.

### DGGE profiles of fungal communities

**Figure 3:**
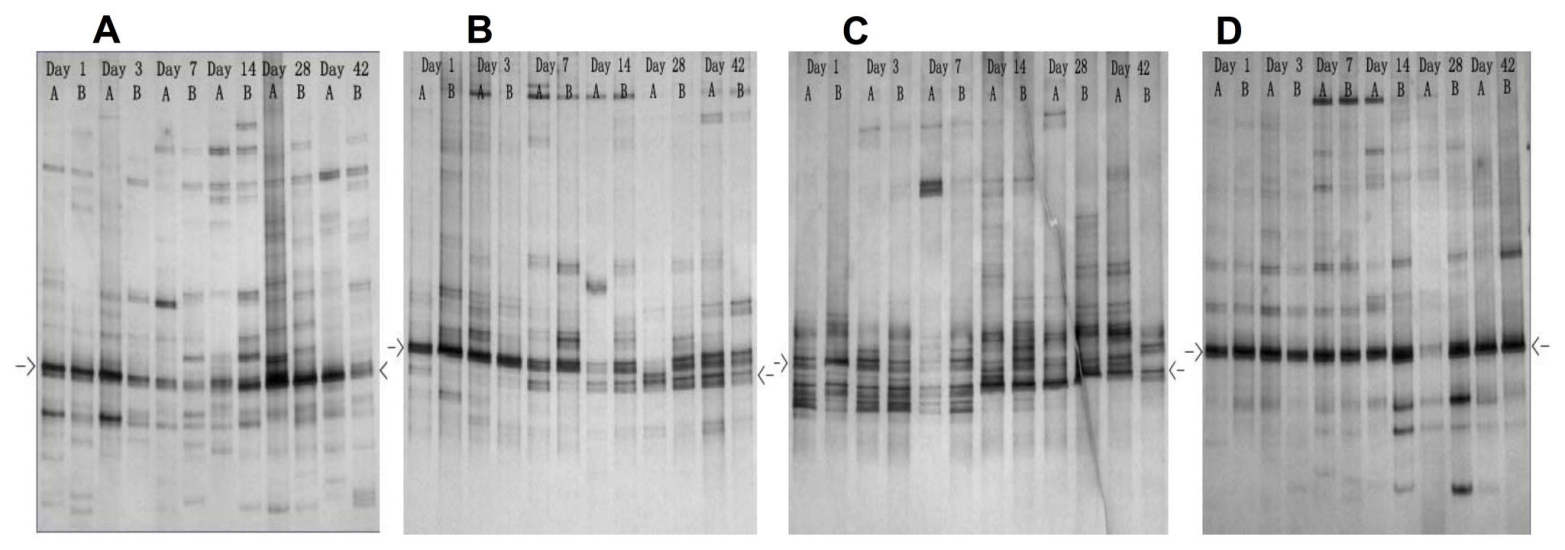
DGGE profiles of fungal 18S rRNA gene amplicons derived from the leaf litter of the plant species A: *Baccaure ramiflora*, B: *Hevea brasiliensis*, C: *Pleioblastus amarus* and D: *Pometi tomentos*. The numbers of days of incubations are indicated. Each sampling day has two replicates of separate incubations denote by A and B. The arrows indicate the bands that have sequences affiliated to *Aspergillus* (day 1) and *Pleosporales* (day 42).

The number of bands of fungal community detected by DGGE analysis was in the range of 15-27, which was less than that of bacteria (Figure 3 and 2C). The richness generally increased towards the end of incubation, except the leaf litter of *Hevea brasiliensis*. Fungal diversity reflected by Shannon’s index based on the DGGE profiles was also lower than that of bacteria and the rising trend was less clear (Figure 2D). The most predominant bands of all four leaf species appeared at the same gradient gel position of the fingerprints (indicated by arrows in Figure 3). These major bands from the first and the 42 days of incubation (except the samples of *Pleioblastus amarus*) were cut, re-amplified and sequenced. The band sequences obtained from days 1 and 42 were affiliated to *Aspergillus* and *Pleosporales*, respectively. The sequences of the three leaf species sampled at the same day had >99% similarity. These bands showed similar DGGE gel positions. On narrower denaturing gradient of 35%-40%, the bands that affiliated to *Aspergillus* located upper than those of Pleosporales, which matched with the total DNA samples (figure not shown). The fungal communities could be roughly divided into two periods, 1-7 and 14-42 days, during the decomposition process, but cluster analysis of the DGGE profiles was unsatisfactory due to the close positioning of the Aspergillus- and Pleosporales-affiliated bands.

### Clone sequences of fungal communities

About 20 fungal 18S rRNA gene clone sequences were obtained from each of the leaf litter species at the first and the last day of incubation (Table 3, phylogenetic trees not shown). Four sequences were affiliated to non-fungi, including three to the genus Pythium and one to Lacane, which were excluded in further analyses. At the beginning of incubation, the fungal communities were dominated by *Aspergillus* and *Cladosporium* that together contributed from 47% up to 100% of the clone libraries. The majority changed to Pleosporales at day 42, except the sample of *Hevea brasiliensis* that still retained 58% of *Aspergillus*. Other major fungi present at the end of the leaf litter incubation were *Cladochytrium*, *Coniochaetales* and *Hypocreale*s. Most sequences fall in a few clusters and within them, the sequences had high similarity of >99%. No significant differences among the fungal communities of all samples were found from both UniFrac distance metrics and P tests.

**Table 3:** Relative abundance and diversity of fungal 18S rRNA gene fragment sequences in various taxa.

	*Baccaura ramiflora L.*		*Hevea brasiliensis*		*Pleioblastus amarus*		*Pometia tomentosa*	
	Day 1	Day 42	Day 1	Day 42	Day 1	Day 42	Day 1	Day 42
*Aspergillus*	45	n.d.	100	58	5	n.d.	76	n.d.
*Aspergillus* cluster 1	40	n.d.	63	42	n.d.	n.d.	47	n.d.
*Aspergillus* cluster 2	5	n.d.	21	16	5	n.d.	6	n.d.
*Aspergillus* cluster 3	n.d.	n.d.	16	n.d.	n.d.	n.d.	n.d.	n.d.
Other *Aspergillus*	n.d.	n.d.	n.d.	n.d.	n.d.	n.d.	13	n.d.
*Pleosporales*	15	47	n.d.	5	29	75	12	95
*Pleosporales* cluster 1	n.d.	37	n.d.	5	n.d.	40	n.d.	90
*Pleosporales* cluster 2	n.d.	n.d.	n.d.	n.d.	15	15	n.d.	n.d.
Other *Pleosporales*	12	10	n.d.	n.d.	14	20	12	5
*Cladosporium*	20	n.d.	n.d.	n.d.	42	n.d.	n.d.	n.d.
*Cladochytrium*	n.d.	26	n.d.	n.d.	n.d.	n.d.	n.d.	n.d.
*Sordariomycetes*	5	11	n.d.	37	5	15	n.d.	n.d.
*Coniochaetales*	n.d.	n.d.	n.d.	37	n.d.	5	n.d.	n.d.
*Hypocreales*	n.d.	11	n.d.	n.d.	5	5	n.d.	n.d.
Other *Sordariomycetes*	5	n.d.	n.d.	n.d.	n.d.	5	n.d.	n.d.
Other fungi	10	16	n.d.	n.d.	19	15	12	5
Number of clones obtained	20	19	19	19	21	20	17	21
Shannon’s diversity index (based on 99% sequence similarity)	1.78	1.79	1.01	1.17	2.19	2.22	0.61	0.75

Fungal taxa that were not detected in the clone library are denoted as n.d. Diversity is calculated based on 99% sequence similarity as operational taxonomy united based on bacterial phyla and 97% sequence similarity as operational taxonomy unit.

## Discussion

### Leaf species effects

The results indicated that leaf species can be considered an influence factor but not so significant one in affecting the microbial decomposer communities, which contrasting our hypothesis that microbial communities involved in decomposition of various tropical leaf litter species are different. Organic carbon and mineral N in the leaves were important resources for microbial growth. In litter with high N content, which resulted in a low C: N ratio, the N required for microbial growth was more abundant than the C required for microbial respiration. On the other hand, in litter with low N content, N became a limiting factor for microbial growth. Therefore, different microbial communities colonized on the litter according to their different C/N requirement [27,28]. Besides C to N ratio, leaf species, which different in other physical and chemical characteristics also have strong influence on decomposition [29,30]. In addition, microbial communities in water environment also have influence on the litter decomposition [31]. Our result may indicate that more specific microbial community associate to leaf species might be present in terrestrial environment, and was replaced by those in stream water. Therefore, leaf quality was considerably more important in regulating the attached microbial community at the initial phage than the later phage. Overall, there were fewer differences among leaf species than might be expected based on differences in leaf properties.

### Microbial diversity and community during decomposition

We observed that both fungal and bacterial diversity increased as predicted during the litter decomposition. The mainly explanation of this was changes of the litter quality: the amount of available carbon as sugars and starch were decreased, while recalcitrant carbon as lignin and phenol were increase during the course of decomposition [32,33]. Couple with the litter quality changes, microbial decomposer various during the litter decomposition. Similar status was found not only in soil environment, but also in water environment. Cox et al. [34] used litterbags containing sterile Scots pine needles inoculated with two species of fungi (*Marasmius androsaceus* and *Trichoderma viride*) in the laboratory. These bags were then placed in the litter layer of a pine plantation for decomposition of litter. Following decomposition over the course of time, they showed that *M. androsaceus*, which can degrade lignocellulose, was initially displaced by other fungal colonizers and was not detected in the litter after 2–3 months, but was re-isolated from the needles after 12 months. In another study, Aneja et al [35] investigate the effect of decomposition site and plant litter species on the colonizing microbial communities, using litter bag in stream, found the importance of litter quality and decomposition site as key factors in their development and succession. 

Sequencing of 16S rRNA clone libraries revealed that, alpha-Proteobacteria represented the main bacterial clade present on leaf litter. The dominance of alpha-Proteobacteria was also found by DAPI and FISH bacterial counts on the leaves disposed in a stream at Ohio [36]. *Sphingomonads* were in majority in this study, which are gram-negative, strictly aerobic chemoheterotrophs, and classified by Enya et al (2007) [37] as leaf colonizers. *Sphingomonas* strains, e.g., *S. asaccharolytica* and *S. oligophenolica*, are capable of degrading aromatic compounds [38]. The alpha-Proteobacterial community present on the leaf litter was diverse. *Hyphomicrobium* possess filaments, which are advantageous in nutrient-uptake and have been reported to associate with sea grass [39]. Species of Rhodobacteriales and Rhizobiaceae have wide range of metabolic capabilities, and Caulobacteriales are chemoorganotrophs. Actinomycetes also represented in leaf degrading bacterial clades, contributing to about 5% of the total bacterial clones. Actinomycetes are gram-positive bacteria (Class Actinobacteria) characterized by high GC content [40], which can significantly contribute to organic matter processing [41]. Actinomycetes, like *Actinokineospora, Streptomyces, Nocardiodes, Pseudonocardia, Nocardia and Micromonospora* [42], have been found on decomposing plant litter. In our study leaves, sequences affiliated to *Microbacterium* and *Catellatospora* were obtained but their roles in leaf litter decay are less clear. Although the leaf litter incubation was conducted under continual aeration, methane-oxidizing bacteria, such as *Methylocystaceae*, *Methylophilus* and *Methylibium*, were present. Strictly anaerobic micro-environment with methane production was probably created in the leaf litter, and the methane was utilized and oxidized by aerobic methanotrophic microbes. 

Sequencing of 18S rRNA clone libraries revealed that fungal diversity increased during decomposition, but in lesser extent than that for bacteria. At the later stage of decomposition, *Pleosporales, Sordariomycetes, Cladosporium* and *Cladochytrium* were present, which were freshwater Ascomycota that may colonize on leaf litter and cause degradation. Sequences that closely related to *Aspergillus restrictus* and *Aspergillus penicilloide* were found to be dominant in the beginning of incubation, which were anamorphic xerophilic mould species and could be potential plant pathogens. Although DGGE and the primer set used in this study have been applied in other similar researches [43], the resolution was unsatisfactory. Main defect was evaluation of community shift, since band positions that corresponded to the major ribo-types affiliating Aspergillus and Pleosporales were close together. Longer gene fragments or higher variable region of ITS would be considered in later studies of this fungal community [44].

### Cultivated vs. DNA based techniques

As with the traditional approach, there are also limitations of the cultivation-based method. First, cultivation does not recover all fungi present, as some fraction of natural microbial communities may not be readily cultivated using conventional means. Second, disparities were observed between the BLAST matches and phylogenetic. DNA-based techniques, such as DGGE, have proved useful for assessing the diversity of aquatic fungi on decomposing leaves and offer the advantage of detecting species from non-sporulating mycelia [18]. Although the assessment of fungal diversity based on spore production may underestimate the number of species, it focuses on reproductive species that are able to disperse, increasing the chance of colonizing new substrata. Overall, greater diversity of fungi was observed via culturing compared to conidia staining; differences between the two leaf species were relatively limited and varied between the methods employed. This further emphasizes the need for assessment of fungal communities using a variety of techniques, especially given that conidia staining alone may not reveal the structure of the entire fungal community. In our study, the cultivation approach reflected the prevalence of mostly non-aquatic hyphomycete taxa while microscopy revealed the aquatic hyphomycete component of the leaf fungal community [45]. Our findings suggest that the non-aquatic hyphomycete taxa need to be explored in more detail to evaluate their role in leaf decomposition.

### Limitation of laboratory incubation

 Microcosms are artificial, simplified ecosystems that are used to simulate and predict the behavior of natural ecosystems under controlled conditions. We used laboratory incubation to carry out our experiment, which have both advantage and disadvantage. The disadvantages are that the real world is not a laboratory and variables change all the time. Also, we might make mistakes in our interpretations of the data and get the cause and effect wrong. The advantages are: psych experiments control for variables, or those things that change all the time in the real world. With this under control, we can then use our results to generalize and perhaps form a theory as to what the results mean (in life). 

## Conclusion

 In conclusion, leaf species properties were controlling factors of the microbial community though not significant, in particular plant species and nitrogen content. We propose that water chemical characteristic rather than litter species were the most important regulating factors for microbial biomass and number of taxa. Both fungal and bacterial community show changes during the decomposition, but different in patterns. 
